# Abdominal distension after eating lettuce: The role of intestinal gas evaluated in vitro and by abdominal CT imaging

**DOI:** 10.1111/nmo.13703

**Published:** 2019-08-11

**Authors:** Elizabeth Barba, Borja Sánchez, Emanuel Burri, Anna Accarino, Eva Monclus, Isabel Navazo, Francisco Guarner, Abelardo Margolles, Fernando Azpiroz

**Affiliations:** ^1^ Digestive System Research Unit University Hospital Vall d'Hebron, Centro de Investigación Biomédica en Red de Enfermedades Hepáticas y Digestivas (Ciberehd) Barcelona Spain; ^2^ Departament de Medicina Universitat Autònoma de Barcelona Bellaterra Spain; ^3^ Departamento de Microbiología y Bioquímica Instituto de Productos Lácteos de Asturias (IPLA), Consejo Superior de Investigaciones Científicas, Asturias (CSIC) Villaviciosa Spain; ^4^ Instituto de Investigación Sanitaria del Principado de Asturias–ISPA Oviedo Spain; ^5^ Kantonsspital Baselland Liestal Switzerland; ^6^ Departamento de Lenguajes y Sistemas Informáticos Universidad Politécnica de Catalunya Barcelona Spain

**Keywords:** abdominal distension, diaphragmatic activity, functional gut disorders, intestinal gas, lettuce

## Abstract

**Background:**

Some patients complain that eating lettuce, gives them gas and abdominal distention. Our aim was to determine to what extent the patients' assertion is sustained by evidence.

**Methods:**

An in vitro study measured the amount of gas produced during the process of fermentation by a preparation of human colonic microbiota (n = 3) of predigested lettuce, as compared to beans, a high gas‐releasing substrate, to meat, a low gas‐releasing substrate, and to a nutrient‐free negative control. A clinical study in patients complaining of abdominal distention after eating lettuce (n = 12) measured the amount of intestinal gas and the morphometric configuration of the abdominal cavity in abdominal CT scans during an episode of lettuce‐induced distension as compared to basal conditions.

**Key Results:**

Gas production by microbiota fermentation of lettuce in vitro was similar to that of meat (*P* = .44), lower than that of beans (by 78 ± 15%; *P* < .001) and higher than with the nutrient‐free control (by 25 ± 19%; *P* = .05). Patients complaining of abdominal distension after eating lettuce exhibited an increase in girth (35 ± 3 mm larger than basal; *P* < .001) without significant increase in colonic gas content (39 ± 4 mL increase; *P* = .071); abdominal distension was related to a descent of the diaphragm (by 7 ± 3 mm; *P* = .027) with redistribution of normal abdominal contents.

**Conclusion and Inferences:**

Lettuce is a low gas‐releasing substrate for microbiota fermentation and lettuce‐induced abdominal distension is produced by an uncoordinated activity of the abdominal walls. Correction of the somatic response might be more effective than the current dietary restriction strategy.

## Graphical abstract

In some patients lettuce produces abdominal distension, which is not related to gas, but rather to an uncontrolled reaction of the abdominal muscles and the diaphragm. Hence, learning to control the abdominal muscles may prevent distension with no need of dietary restrictions.

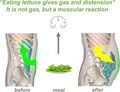

**Key Points**

- Some patients complain that eating lettuce gives them gas and abdominal distention; however, there is no evidence in support of this assertion.
- Lettuce is a low gas‐releasing substrate for microbiota fermentation both in vitro and in vivo; lettuce‐induced abdominal distension is a somatic expression with diaphragmatic contraction and protrusion of the anterior abdominal wall.
- Identification of the conditioning mechanism of this response would help to develop a more specific treatment than the current dietary restriction strategy.

## 1 INTRODUCTION

Some patients complain that eating lettuce gives them gas and abdominal distention. Our aim was to determine to what extent the patients' assertion is sustained by evidence.

The food ingested undergoes a process of digestion in the oral cavity, stomach, and small intestine before the resulting chime enters the colon. Food residues within the colon serve as substrates for microbiota metabolism. Fermentation of food residues by microbiota releases gas at an amount that depends on the type of substrate; gas production is large with fermentable carbohydrates, and low with proteins and fat.^1^

The abdominal wall actively adapts to its content by a tight regulation of its muscular activity.^2^, ^3^ Considering these two elements, ie wall and content, abdominal distension in patients may be related to either an increase in abdominal content, eg intestinal gas accumulation, or to a dyssynergia of the abdominal walls and redistribution of normal content; the latter is a conditioned response that can be corrected by behavioral treatment.^4^, ^5^, ^6^

To address our aim, a two‐phase study was performed. An in vitro study measured the amount of gas produced during the process of microbiota fermentation of lettuce, as compared to a high gas‐releasing substrate, a low gas‐releasing substrate and a nutrient‐free negative control. Beans were used as a high gas‐releasing substrate by human colonic microbiota demonstrated both in vivo^7^ and in vitro,^8^, ^9^ and cow meat, with a very low content of non‐digestive fermentable polysaccharides, as a potential low gas‐releasing substrate. After a pre‐processing mimicking cooking and oro‐gastro‐intestinal digestion, each foodstuff was incubated with a preparation of human colonic microbiota, and the amount of gas released by fermentation was measured. In a clinical phase, patients complaining of abdominal distention after eating lettuce were studied, measuring the amount of intestinal gas and the morphometric configuration of the abdominal cavity in abdominal CT scans during an episode of lettuce‐induced distension as compared to basal conditions.

## 2 MATERIAL AND METHODS

### 2.1 In vitro study: Gas production during fermentation of specific foodstuffs by human colonic microbiota

Gas production was measured in batch fecal microbiota preparations from healthy donors cultivated in the presence of in vitro digested foods.

#### 2.1.1 Test foodstuffs and preparation

Four preparations were tested: iceberg lettuce (*Lactuca sativa*), cow meat (Bos taurus; sirloin) and broad beans (*Phaseolus vulgaris*, variety “Granja Asturiana”), and a nutrient‐free negative control (phosphate‐buffered saline, PBS). To reproduce cooking conditions, 50 g cow meat was boiled for 20 minutes in 1 L of water, and 50 g broad beans were boiled for 30 minutes in 1 L of water leaving them to soak in the same water at room temperature for another 30 minutes; fresh lettuce was used without any cooking treatment.

#### 2.1.2 In vitro digestion

In order to mimic the digestive process, a standardized static in vitro digestion method was used following international guidelines 10; the composition of Simulated Salivary Fluid (SSF), Simulated Gastric Fluid (SGF), and Simulated Intestinal Fluid (SIF) has been reported in detail.^10^

##### Oral digestion

Samples (5 g) of each foodstuff and the nutrient‐fee control were homogenized with 5 mL of prewarmed SSF at 37ºC in a compact masticator paddle blender (Reference code 100085/058; IUL Instruments, Barcelona, Spain) during 1 minute, and incubated for 2 minutes at 37ºC; SSF contained 1.5 mmol/L CaCl_2_ and 75 U/mL human salivary α‐amylase Type IX‐A (ref. A0521; Sigma‐Aldrich).

##### Gastric digestion

The resulting oral bolus was mixed with 10 mL of prewarmed SGF, the mix was adjusted to pH 3 with HCl and incubated for 2 hours at 37ºC with mild shaking every 30 minutes; SGF contained 0.15 mmol/L CaCl_2_ and 2000 U/mL porcine pepsin (ref. P7000; Sigma‐Aldrich).

##### Intestinal digestion

After the gastric phase, 20 mL of prewarmed SIF was added to the gastric chime, the pH was adjusted to 7.0 with NaOH and the resulting mixture was incubated for 2 hours at 37ºC with mild shaking every 30 minutes; SIF contained 0.6 mmol/L CaCl_2_ and 100 U/mL porcine pancreatin (ref. P7545; Sigma‐Aldrich) and 10 mmol/L porcine bile salts (ref. B8631; Sigma‐Aldrich).

After the simulated digestion, the samples of the four preparations (the three digested foodstuffs and the nutrient‐free negative control) were divided in three different aliquots and immediately stored at −80ºC.

#### 2.1.3 Colonic microbiota preparations

Fecal samples were obtained from three newly recruited donors without known diseases, gastrointestinal symptoms, antibiotic intake in the past 6 months and with normal bowel habit. Fecal slurries of each sample were prepared, as follows: An aliquot (4 g) of each fecal sample was thoroughly mixed in a total volume of 40 mL PBS during 5 minutes using a Lab Blender 400 Stomacher (ref. 432‐0161; Seward Medical) and A modified 2× basal medium was prepared by adding 4 g/L peptone water, 4 g/L yeast extract, 0.2 g/L NaCl, 0.08 g/L K_2_HPO_4_, 0.08 g/L KH_2_PO_4_, 0.02 g/L MgSO_4_, 0.02 g/L CaCl_2_.2H_2_O, 4 g/L NaHCO_3_, 5 g/L L‐cysteine‐HCl, 4 mL/L Tween‐80, and 0.1 g/L haemin.^11^ With each individual's microbiota, four slots were prepared by mixing 40 mL of the 2× basal medium with 8 mL fecal slurries and 20 mL of sterile dH_2_O.

#### 2.1.4 Preparation of fecal cultures

Each slot of microbiota preparation was mixed with 12 mL of one of the previously digested preparations: lettuce, ie the test meal, meat, ie the low gas‐producing control, beans, ie the high gas‐ producing control, and the nutrient‐free negative control. Gas production by each of the three independent fecal microbiotas fed with the different substrates (total 12 preparations) was monitored using the ANKOM^RF^ Gas Production System (ANKOM Technology) in an anaerobic workstation (MG500 Anaerobic Workstation, Don Whitley Scientific) at 37ºC during 30 hours, under a H_2_:CO_2_:N_2_ (1:1:8) atmosphere. Gas production in each preparation was continuously monitored by measuring the increment in pressure.

### 2.2 Clinical study

#### 2.2.1 Participants

Twelve women (age range 24‐58 years) who complained of abdominal distension after eating lettuce participated in the study. Only patients who reported episodes of visible distension related to ingestion of lettuce in contrast to basal periods with mild or no distension were included in the study. The entry criteria required lettuce as the primary offending foodstuff, regardless that other green leafy vegetables could have a deleterious effect as well. All patients had a functional disorder diagnosis based on Rome III criteria 12: 8 functional bloating (7.1 ± 0.5 bowel movements per week; 3.2 ± 0.3 score on the Bristol stool form scale) and four constipation‐predominant irritable bowel syndrome (2.2 ± 0.3 bowel movements per week, 2.0 ± 0.4 Bristol score). Symptom duration was 6 ± 4 years.

The study protocol had been previously approved by the Institutional Review Board of the University Hospital Vall d’Hebron, and all subjects gave their written informed consent to participate in the study. This study is an exploratory arm of a larger study (ClinicalTrials.gov NCT01205100) involving a subset of patients who fulfilled the inclusion criteria described above, and hence, no power calculation was performed; data on abdominal CT imaging have not been previously published.

#### 2.2.2 Experimental procedure

After having recognized lettuce as the reproducible offending meal, ie patients knew that eating lettuce would give them abdominal distension, two visits were scheduled within a 2‐day interval: patients were instructed to report in the laboratory (a) during fasting when they felt minimal or no abdominal distension (basal conditions), (b) and within 1 hour after eating the offending foodstuff (lettuce salad dressed at taste). During each condition (basal and distension) the subjective sensation of abdominal distension and abdominal girth were measured, and a CT scan was taken, as detailed below. Afterward, patients were offered a biofeedback treatment to correct abdominal distension.

#### 2.2.3 Subjective sensation of abdominal distension

The patient's subjective sensation of abdominal distension was measured on a 6‐score graphic rating scale graded from 0 (no distension) to 6 (extremely severe distension).

#### 2.2.4 Girth measurement

The method has been previously described and validated in detail.^13^, ^14^, ^15^, ^16^, ^17^ Briefly, a non‐stretch belt (48‐mm wide) with a metric tape measure fixed over it was placed over the umbilicus. The overlapping ends of the belt were adjusted carefully by two elastic bands to maintain the belt constantly adapted to the abdominal wall. Girth measurements were taken with the subjects breathing quietly as the average of inspiratory and expiratory determinations over three consecutive respiratory cycles without manipulation of the belt‐tape assembly by the investigator. In the first measurement (basal conditions or distension episode), the location of the belt was marked on the skin for subsequent measurements.

#### 2.2.5 Abdominal CT imaging and analysis

Abdominal CT scans were performed blindly with the operator unaware of the condition (basal or distension) of the patient. The CT scan during lettuce‐induced distension was taken 1 hour after ingestion. Scanning was performed with a helical multislice CT scanner (Somatom Definition AS, Siemens Medical Solutions), exposure 120 kV and 50 mAs, using the available dose reduction options (tube current modulation); 2‐mm section thickness and 1.5‐mm interval reconstruction. Images were obtained in the supine position during a single breath‐hold at the end of expiration. No oral or intravenous contrast medium was administered.

Analysis of CT images was performed blindly. Morphovolumetric analysis of CT images was performed using an original software program specifically developed in our laboratory and previously described in detail.^6^, ^18^

##### Gas volume in the gut

To measure the volume of gas within the gut, images were filtered with a user‐defined threshold to separate gas from tissues.

##### Abdominal morphometric analysis

Abdominal perimeter was measured by averaging the perimeter of the abdominal surface measured in 10 axial slices 4 mm apart at the level of the umbilicus; at each site, the perimeter was measured as the length of a polyline (series of connected segments) following the body contour. Antero‐posterior abdominal diameter was measured as the distance (in the antero‐posterior axis) between the anterior aspect of the vertebral bodies and the midline surface of the anterior abdominal wall; the average of the values measured at six levels (L_1_ to S_1_) was calculated in each subject. Position of the diaphragm was measured as the distance (in the vertical axis) between the left diaphragmatic dome and the cranial end‐plate of the twelfth thoracic vertebra (T_12_).

Total abdominal volume (gas plus liquids and solids) was measured as the body volume between a cranial plane (tangential to the diaphragmatic domes and perpendicular to the vertebral spine) and a caudal plane (defined by bony structures in the pelvis) subtracting the volume corresponding to the lungs and the heart.

#### 2.2.6 Biofeedback treatment

Patients received EMG‐guided training during the three treatment sessions on separate days within a 2‐week period, as described before.^5^, ^19^ In brief, patients were trained to control the activity of the abdomino‐thoracic muscles under visual control of EMG recordings displayed on a monitor. Specifically, they were instructed to reduce the activity of intercostal muscles and the diaphragm, while increasing the activity of the anterior abdominal muscles. After each biofeedback session, patients were instructed to perform the same exercises daily at home for 5 minutes before and after breakfast, lunch, and dinner. By the end of treatment, the subjective sensation of abdominal distension and abdominal girth were measured.

### 2.3 Statistical analysis

Results of the “in vitro” study are presented as the mean (±SE) of the three biological replicates for each of the three foodstuffs and the control solution. Mean values or grand means for repeated observations on the clinical study, ie treatment sessions of the parameters measured (±SE) were calculated in each group of subjects. Normality was tested by the Kolmogorov‐Smirnov test. Comparisons of parametric, normally‐distributed data were made by the paired Student's *t* test; otherwise, the Wilcoxon signed‐rank test was used.

## 3 RESULTS

### 3.1 In vitro gas production

**Figure 1:**
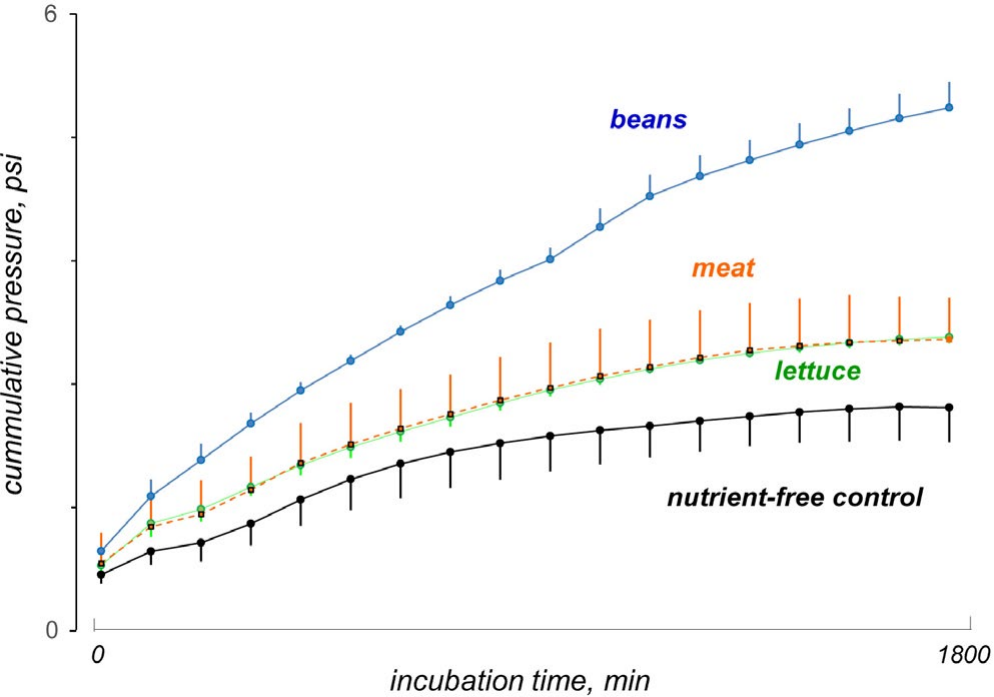
Gas production by microbiota fermentation in vitro. The three predigested foodstuff and the nutrient‐free control were incubated with preparations of human colonic microbiota (n = 3). Gas production by microbiota fermentation of lettuce was similar to that of meat (a low gas‐releasing substrate), 78 ± 15% lower than that of beans (a high gas‐releasing substrate; *P* < .001) and 25 ± 19% higher than with the nutrient‐free control (*P* = .05)

Gas production by microbiota fermentation of lettuce, the test product, was similar than that of meat, the low gas‐producing control. Gas production by both preparations was significantly lower than that during fermentation of beans, the high gas‐producing control, and higher than with the negative nutrient‐free control (Figure 1).

### 3.2 Clinical study

#### 3.2.1 Abdominal bloating and distension

When patients complained of distension after ingestion of lettuce, the subjective sensation of abdominal distension (4.5 ± 0.2 score) was significantly higher than during basal condition (2.0 ± 0.4 score; *P* < .001). Furthermore, objective measurement of abdominal girth by tape measure also detected significant differences compared to basal conditions (35 ± 3 mm larger than in the basal session; *P* < .001).

#### 3.2.2 Content of gas within the digestive tract 

**Figure 2:**
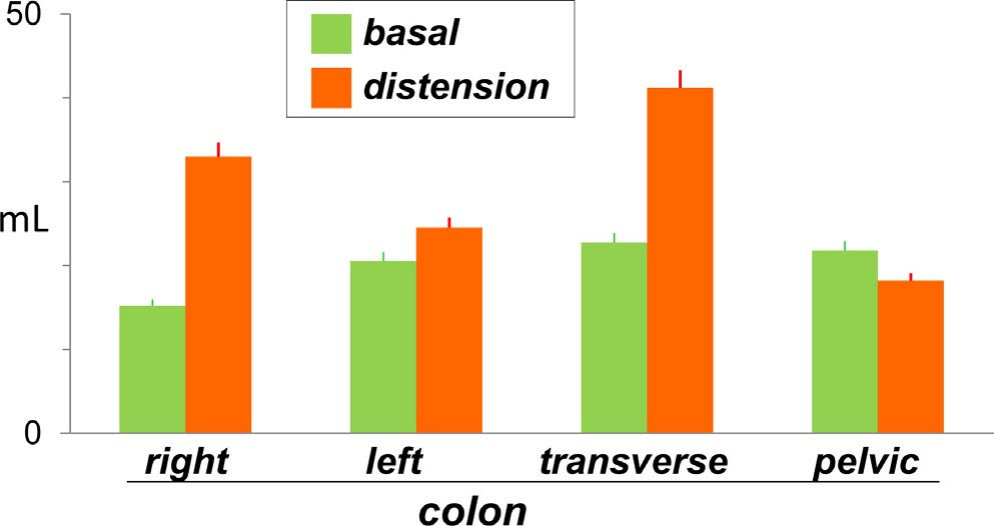
Distribution of gas in the colon. During a lettuce‐induced episode of abdominal distension, colonic gas content tended to be higher, but not significantly, than during basal conditions. To note, the absolute differences were small and would hardly account for the abdominal distension

In the CT scans taken during basal conditions, the volume of gas in the colon was 85 ± 16 mL. Gas was evenly distributed along the colon within the different compartments (Figure 2). During abdominal distension, the gas volume in the colon tended to increase (by 39 ± 4 mL), but the difference was not statistically significant (*P* = .071; Figure 2). The volume of gas in the small bowel (24 ± 14 mL in the basal scan) and in the stomach (12 ± 2 mL basal) also tended to increase (increase by 12 ± 9 mL and by 41 ± 10 mL, respectively) but the differences were not statistically significant (*P* = .089 and *P* = .111, respectively).

#### 3.2.3 Mechanism of abdominal distension

##### Abdominal morphometric analysis

**Figure 3:**
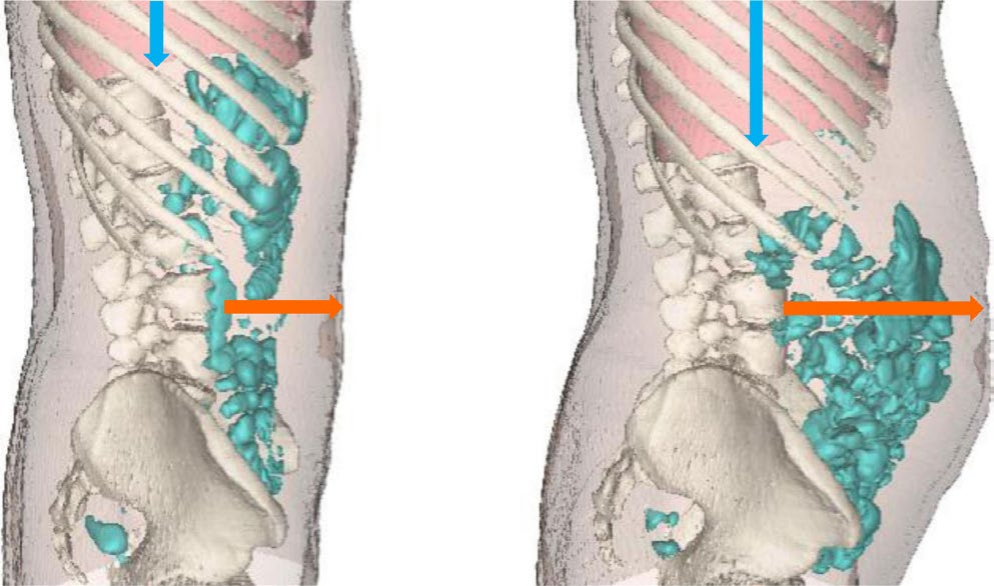
Abdominal CT image in a patient during basal conditions and during an episode of lettuce‐induced distension. Note that abdominal distension is associated to a diaphragmatic descent (blue arrow) and anterior wall protrusion (orange arrow) without substantial increase of intestinal gas

Morphometric analysis of abdominal CT images showed that abdominal distension was associated with a marked increase in abdominal girth (20 ± 2 mm greater than in basal scans; *P* < .001), confirming the tape measurements, and in the antero‐posterior abdominal diameter (16 ± 5 mm greater than in the basal scan; *P* = .011). Abdominal distension was associated with a caudal displacement of the diaphragm, measured as a decrease in the distance from the diaphragmatic dome to T_12_ (7 ± 3 mm descent; *P* = .027; Figure 3). The difference in total intraabdominal volume (between the fasting basal scan and the postprandial distension scan was 835 ± 187 mL.

### Reponses to biofeedback treatment

Under the visual guidance provided by the EMG signal, all patients were able to effectively control their abdominal muscular activity. Treatment was associated with a significant improvement in the subjective sensation of abdominal distension (from 4.5 ± 0.2 score to 2.2 ± 0.3 score; 50 ± 7% reduction; *P* < .001) and decrease in girth (by 50 ± 2 mm; *P* < .001).

## 4 DISCUSSION

Our data indicate that when patients complain of abdominal distention produced by gas after eating lettuce, abdominal distention is real, but their interpretation is incorrect. Indeed, abdominal distension is not produced by intestinal gas, but by an uncoordinated activity of the abdominal walls, with contraction of the diaphragm, caudal displacement of abdominal content and protrusion of the anterior abdominal wall. The response to behavioral treatment suggests that this is a conditioned response. Hence, our exploratory study shows that: (a) lettuce may produce objective abdominal distension, (b) distension is not related to gas, and (c) it is related to a somatic behavioral response. Beyond these main pieces of reasonable evidence, our study does not elucidate the specific factors in lettuce and the underlying mechanisms leading to the abnormal somatic response.

After eating lettuce, gas content in the colon increased but to an extent that would hardly account for an episode of visible abdominal distension. The increase in gas content observed was probably related to the arrival into the colon of complex carbohydrates resistant to small bowel digestion. The amount of colonic gas and the intraluminal distribution of the gaseous mass, both during basal conditions as well as after eating lettuce, was within the normal range observed in healthy subjects during fasting and after a regular meal.^18^, ^20^, ^21^, ^22^

The clinical data are supported by the in vitro study. Fermentation of lettuce by colonic microbiota in vitro produced a similar amount of gas as meat, with a low content of carbohydrates, and significantly less than beans, high in fermentable carbohydrates. During physiological conditions in humans, fermentation of meal residues by colonic microbiota can be monitored by measuring colonic gas production.^23^, ^24^, ^25^, ^26^ Gas production peaks after ingestion with the arrival into the colon of fresh substrates escaping small bowel absorption; subsequently gas production declines,^24^ but still the fecal material evacuated contains fermentable residues. Indeed, the presence of fermentable residues in the fecal samples may explain the production of gas in vitro with the nutrient‐free negative control. The static in vitro digestion method, recommended by international consensus,^10^ does not reproduce the absorption of nutrients that normally occurs in the small bowel, and hence, gas production after eating lettuce in vivo would be even smaller, because digestible carbohydrates would not arrive into the colon.

Previous studies in healthy subjects using abdominal CT imaging combined with EMG of the abdominal walls showed that the anterior wall and the diaphragm, adapt their muscular tone to the intraabdominal content. Increases of intraabdominal content, induce an abdominal accommodation response, featuring a relaxation and upward displacement of the diaphragm with cephalad expansion of the abdominal cavity and limited impact on the anterior abdominal wall.^3^, ^17^, ^27^, ^28^ The present study shows that abdominal distension after eating lettuce in our patients is produced by a paradoxical contraction of the diaphragm with caudo‐ventral redistribution of abdominal content. This mechanism of abdominal distension has been also detected in other patients with episodic abdominal distension not specifically related to meals.^5^, ^6^ To treat this condition, we developed in our laboratory a biofeedback technique that allows patients to control the activity of the abdominal walls.^5^, ^19^ The fact that our patients learned to modulate the activity of the abdominal walls, and thereby prevented lettuce‐induced distension, suggests that the latter is a behavioral response under volitional control.

We wish to acknowledge that this pilot study included a small sample size and did not include proper controls for the specificity of lettuce as the offending foodstuff. Furthermore, we can only speculate on the mechanisms of conditioning, ie what in lettuce and by which mechanism triggers the abnormal somatic response. An interesting study by the Nottingham group has recently shown that lettuce increases water content in the small bowel, possibly due to irritant latex‐like lactucins inducing secretion.^29^ In our study, the increment in total intraabdominal contents in the postprandial distension scan was within the normal range and would normally elicit a proper abdominal accommodation. However, in sensitive individuals either the irritant component in lettuce or even a relatively small increase small bowel water content not detectable by CT imaging, may release the abnormal response. Alternatively, or additionally, lettuce‐induced distension may be related to cognitive/affective factors: anticipation may trigger the behavioral response, ie conditioned patients might become distended if they believe they will. In general, stress is reported as a frequent triggering factor, which supports the potential role of the mental sphere.^30^ Nevertheless, why these patients in the first place learned this abnormal behavior is not known.

The straightforward management of this condition relays on dietary restriction avoiding the offending foodstuff, but this is cumbersome and in the long run may result unpractical. Disentangling the conditioned behavior may be a better strategy. Currently, deconditioning of these patients may be achieved focussing on the somatic mechanism of distension by biofeedback; however, the identification of the specific origin of conditioning and the triggering factor would allow a more selective and effective alternative.

## CONFLICT OF INTEREST

No competing interests declared.

## AUTHOR CONTRIBUTIONS

EBarba. Clinical study management, conduction of experiments. BS. Study design, in vitro studies, data analysis, manuscript preparation. EBurri. Design of the original clinical study. AA. Clinical study design, supervision of studies, data analysis. EM. Adaptation of CT analysis program. IN. Adaptation of CT analysis program. FG. Data interpretation, manuscript revision. AM. Study design, in vitro studies, data analysis, manuscript revision. FA. Study design, data interpretation, manuscript preparation.
